# Definition and use of “valid” district level vaccination coverage to monitor Global Vaccine Action Plan (GVAP) achievement: evidence for revisiting the district indicator

**DOI:** 10.7189/jogh.08.020404

**Published:** 2018-12

**Authors:** David W Brown

**Affiliations:** Brown Consulting Group International, LLC, Cornelius, North Carolina, USA

## Abstract

**Background:**

The Global Vaccine Action Plan (GVAP) Monitoring and Accountability Framework includes an indicator to reach 90% national vaccination coverage and 80% vaccination coverage in every district or equivalent administrative unit with three doses of diphtheria-tetanus-pertussis containing vaccines (DTP) across all 194 country signatories to GVAP by 2020. Assessment of progress against the district indicator component requires GVAP defined “valid” coverage. GVAP defines district coverage “valid” if the WHO and UNICEF estimate of national immunization coverage for DTP3 in the most recent year is 1) ≥90%, or 2) is identical to the reported national administrative coverage for DTP3 (regardless of coverage level). We draw attention to the potential disconnect that currently exists between GVAP vaccination coverage indicators and the practical capacity to monitor progress against those indicators.

**Methods:**

We obtained national and aggregated district coverage data for the third dose of DTP containing vaccine (DTP3) for 194 countries for 2016 from publicly available databases maintained by the World Health Organization (WHO). We reviewed district line lists of coverage data for 96 countries for which district line lists of DTP3 coverage were available and categorized the district coverage values using the same groupings used by the GVAP Monitoring Framework. In doing so, we also tracked the number of districts with reported coverage >100%.

**Results:**

In 2016, at least one district with DTP3 coverage value >100% was reported in the line lists of 76 of the 96 countries. Agreement in district coverage categories across each of five coverage groupings (<50%, 50-79%, 80-89%, 90-94%, ≥95%) was observed in 43 of the 96 countries. In 2016, 46 of 194 countries were classified as achieving both national DTP3 coverage ≥90% and DTP3 coverage ≥80% in every district, thereby achieving the GVAP target. Among these 46 countries, 22 countries reported district line-listing coverage data. We identified 10 of the 22 countries that reported at least one district with DTP3 coverage >100% ranging from 8% of districts in Sri Lanka to 97% of districts in Bangladesh. Seven countries reported at least 25% of the total districts had DTP3 coverage >100%.

**Conclusions:**

The observations of disparate district coverage from a subset of countries reporting district line lists of coverage data are a concern for the current GVAP approach ascribing the achievement of “valid” district coverage data. Our review of district line lists of coverage data does not support a current GVAP assumption that the district coverage values ≥80% fall between 80% and 100% (inclusive). We hope these results spur a review of the current approach to assess the GVAP coverage target of ≥90% national DTP3 coverage and ≥80% DTP3 coverage in all districts.

In 2012, all 194 World Health Organization (WHO) Member States at the Sixty-fifth World Health Assembly adopted the Global Vaccine Action Plan (GVAP), a framework for achieving the ambitious vision of the Decade of Vaccines (2011-2020) – to realize “…a world in which all individuals and communities enjoy lives free from vaccine-preventable diseases” [1]. An explicit goal of the GVAP Monitoring and Accountability Framework is to meet vaccination coverage targets in every region, country and community. The indicators for this goal include:

- to reach 90% national coverage and 80% coverage in every district or equivalent administrative unit with three doses of diphtheria-tetanus-pertussis containing vaccines (DTP) by 2020; and
- to reach at least 90% national coverage and 80% coverage in every district or equivalent administrative unit with all vaccines in national programmes, unless otherwise recommended, by 2020 [2].

We applaud the global effort and share GVAP goals, but draw attention to the potential disconnect that currently exists between GVAP vaccination coverage indicators and the practical capacity to monitor progress against those indicators.

At present, only the first indicator can be conceivably monitored using the data countries report to WHO and the United Nations Children’s Fund (UNICEF). Through an annual immunization system performance data collection exercise [3], WHO and UNICEF jointly collect nationally reported coverage data for most, though perhaps not all, vaccines in national immunization schedules across all 194 Member States. District level coverage data are only collected for DTP containing vaccine and measles containing vaccine.

In addition to information gaps, data quality is a recognized challenge of monitoring vaccination coverage [4,5], and consequently, progress against endorsed global targets. GVAP indicators currently source national level coverage from WHO and UNICEF Estimates of National Immunization Coverage (WUENIC) that take into consideration coverage data of varied and unknown quality that are reported by national immunization programmes [6-8]. The GVAP district level coverage indicator is sourced from nationally reported aggregate summaries of coverage for the third dose of DTP containing vaccine and measles containing vaccine on Sheet 6: Indicators of the WHO/UNICEF Joint Reporting Form on Immunization (JRF) [3]. District level coverage data reported by countries are not well understood – documentation and metadata are often lacking for the various administrative recording and reporting systems that exist across the 194 countries that are sent through the standard questionnaire each year. The district level coverage data used in monitoring the GVAP indicator are derived from line lists of district coverage that each country aggregates, summarizes and reports to WHO and UNICEF in a form similar to **Table 1**.

**Table 1:** WHO/UNICEF Joint Reporting Form-6 – immunization System Indicators: district coverage reported for routine immunization services in 201x

	Coverage <50%	Coverage 50–79%	Coverage 80–89%	Coverage 90–94%	Coverage ≥95%	Number of districts not reporting
Number of districts with DTP3 coverage in each range						
Number of surviving infants in these districts						

Perhaps to account for the unknown quality of district level coverage data, the GVAP Monitoring and Accountability Framework adds a conditional requirement before district data (if available) are considered. Specifically, district level data are only considered if the WHO and UNICEF estimate of national immunization coverage for DTP3 in the most recent year is 1) ≥90%, or 2) is identical to the reported national administrative coverage for DTP3 (regardless of coverage level). If either of these conditions is met, then the district level coverage reported in the JRF is defined as “valid” and district coverage values across coverage categories 80-89%, 90-94% and ≥95% are aggregated to assess progress against the GVAP indicator – whether all districts achieved DTP3 coverage ≥80%.

We used available district line lists of vaccination coverage data reported to the WHO in 2017 to 1) identify possible inconsistencies with the current GVAP Monitoring and Accountability Framework approach for classifying district coverage data as “valid” and 2) identify potential conflicts in the data being used to assess whether GVAP district level coverage targets are achieved.

## METHODS

We obtained the WUENIC for the third dose of DTP containing vaccine (DTP3) for all 194 member states that were signatories to the GVAP for 2016 (the most recent year of data available at the time of this analysis) from online databases provided by the WHO [9]. Detailed descriptions of the WUENIC production are provided elsewhere [6-8]. We also downloaded nationally reported district level aggregate coverage summaries for DTP3 reported to WHO and UNICEF on the JRF [3], a process described in the methods section of Brown and Gacic-Dobo [10]. In addition, we requested and received line listing district level coverage data for 2016 from the WHO [11].

Briefly, in 2017, the WHO requested all countries to report subnational (first and second administrative level or equivalent unit) administrative data (including the number of children vaccinated, the target population size and computed coverage value) for the first and third doses of DTP containing vaccine and for the first dose of measles containing vaccine. A total of 140 countries shared line listings of their subnational coverage data. We identified 96 countries (accounting for 58% of the estimated global birth cohort for 2016 by the United Nations Population Division) that submitted coverage data for the second subnational administrative level, ie, district, for DTP3 for 2016.

After reviewing each country’s data, we categorized the district coverage values using the same categories, as shown in **Table 1**, used by countries when completing the JRF. We added a category to track the number of districts with coverage >100%, a category that is currently not included in the WHO/UNICEF data collection form. We compared the reported aggregated district counts from our categorization of coverage across each of the five DTP3 coverage categories (<50%, 50-79%, 80-89%, 90-94%, ≥95%) to those submitted by countries to WHO and UNICEF in Sheet 6 of the JRF with our categorization results based on the district line list of DTP3 coverage data.

## RESULTS

**Figure 1:**
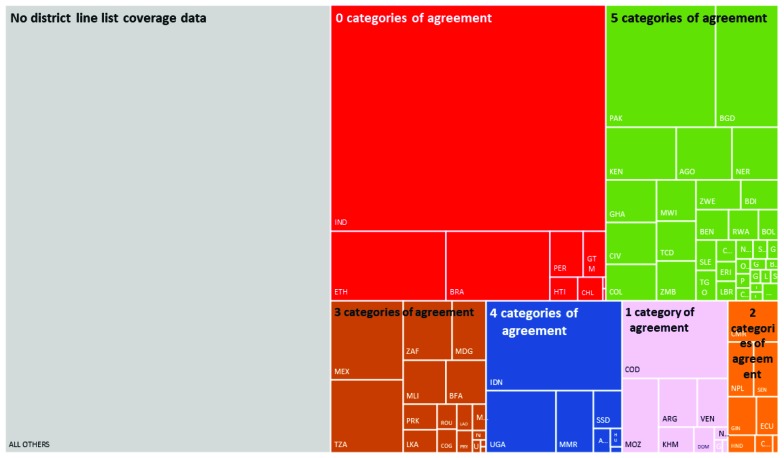
Tree mapping for 96 countries and the agreement observed between comparisons of reported aggregated district counts and a re-categorization of district line lists of coverage. Note: Shaded areas in the tree map are proportional to each country’s 2016 estimated global birth cohort. Countries are indicated on the tree map according to their ISO-3 code (available online at United Nations Trade Statistics Knowledgebase, https://unstats.un.org/unsd/tradekb/knowledgebase/country-code). A listing of countries included in the tree map by category of agreement is available in Appendix S1 in **Online Supplementary Document(Online Supplementary Document)**.

Across the 96 countries for which district line lists of coverage data were available, there was agreement in aggregated district counts for all five coverage categories in 43 countries and no agreement across any of the coverage categories in nine countries (**Figure 1**). At least one district DTP3 coverage value >100% was reported in the line lists of 76 of the 96 countries sharing such data.

**Figure 2:**
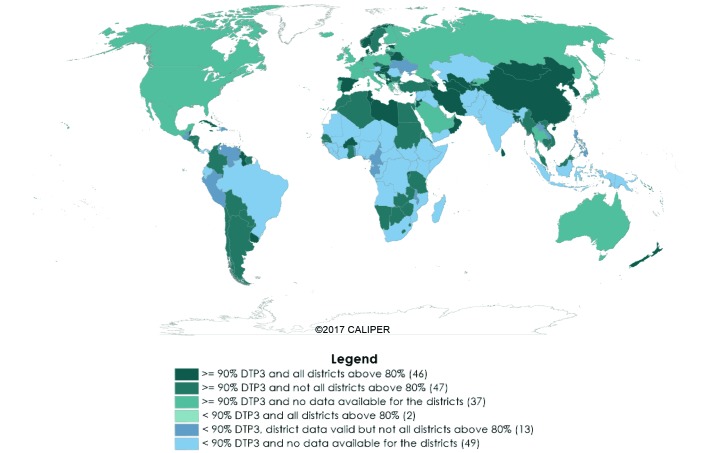
Global Vaccine Action Plan coverage indicator achievement for 194 countries, 2016. Map Disclaimer: The designations employed and the presentation of the material in this publication do not imply the expression of any opinion whatsoever on the part of BCGI LLC concerning the legal status of any country, territory, city or area or of its authorities, or concerning the delimitation of its frontiers or boundaries. Dotted and dashed lines on maps represent approximate border lines for which there may not yet be full agreement.

In the GVAP 2017 report [2], 108 of 194 countries were classified as having “valid” district level data (**Table 1**). In the remaining 86 countries, district data were either not valid (n = 38) or data were not reported (n = 48). A total of 130 countries achieved national DTP3 coverage ≥90% in 2016; 93 of these countries were identified as having “valid” district coverage data. Among the 93 countries with “valid” district coverage, 46 countries were classified as achieving both national DTP3 coverage ≥90% and DTP3 coverage ≥80% in every district, thereby achieving the GVAP target (**Figure 2**; **Table 1**).

Of the 108 countries with GVAP defined “valid” district level data, 63 shared district line listing data for the 2016 calendar year (**Table 2**). Fifty-two of the 63 countries achieved national DTP3 coverage ≥90% by GVAP appraisal. Across the 63 countries with GVAP valid district data and with reported district line-listing coverage data, 25 countries reported at least one district with DTP3 coverage <50%; among these 25 countries, the mean and median percentage of districts in each country with coverage <50% was 6% and 5% of districts, respectively. A total of 40 of the 63 countries reported at least one district with DTP3 coverage between 50-79%, and 45 of the 63 countries reported line-listing district DTP3 coverage >100% for at least one district. The percentage of districts in each of the 45 countries reporting district DTP3 coverage >100% ranged from 5% in Myanmar to 97% in Bangladesh (mean: 32% of total districts; median: 30%; 25% quartile (Q25): 13%; Q75: 47%). Twenty-three countries reported at least one district with DTP3 coverage <50% and at least one district with DTP3 coverage >100%; 16 of these 23 countries had estimated national DTP3 coverage ≥90% for 2016 (ie, and thus GVAP valid).

**Table 2:** National DTP3 coverage, GVAP-defined valid district-level coverage data and reported district line listing data for 2016

	National DTP3 coverage*		
	**<90%**	**≥90%**	**Overall**
GVAP valid district data	15	93	108
≥80% coverage in all districts	2	46	48
Not all districts achieve 80% coverage	13	47	60
Reported line listing district data	11	52	63
+ at least one district reporting <50% coverage	7	18	25
+ at least one district reporting >100% coverage	8	37	45
District data not valid or no report	49	37	86
Overall	64	130	194

DTP3 – diphtheria-tetanus-pertussis, GVAP – Global Vaccine Action Plan

*National DTP3 coverage based on WHO and UNICEF estimates of national immunization coverage, 2016 revision (completed July 2017). Valid district data reported by national immunization programmes to WHO and UNICEF on through the Joint Reporting Form on Immunization. Values reported in the table are numbers of countries.

Of the 46 countries that were reported in the 2017 GVAP report as achieving national level DTP3 coverage ≥90% and coverage ≥80% in every district, 22 countries reported district line-listing coverage data. None of these 22 countries reported any districts with DTP3 coverage <80% in the line-listing data file; however, 10 countries reported at least one district with DTP3 coverage >100%. In Sri Lanka, 8% of districts were reported with DTP3 coverage >100%. In Bangladesh, as noted above, 97% of districts were reported with DTP3 coverage >100%. In addition to Bangladesh, seven countries reported at least 25% of the total districts had DTP3 coverage >100% – these include, Bhutan, 25% of districts; Belize, 33%; St Vincent and the Grenadines, 44%; Cuba, 47%; Rwanda, 53%; Jamaica, 54%; Burkina Faso, 60%. The remaining 12 countries (Albania, Azerbaijan, Hungary, Macedonia, Norway, Gambia, Sao Tome and Principe, Maldives; Oman, People’s Republic of Korea, Brunei, Uruguay), all reported district level DTP3 coverage values ranging between 80% and 100%.

## DISCUSSION

Although we have no available evidence to suggest the reported coverage data for districts with coverage values between 0% and 100% are any more, or less, likely to accurately reflect programme performance than that for districts with coverage values >100%, the observations described here perhaps should give pause and stimulate further discussion about the assumptions inherent in the assessment of subnational coverage performance in the GVAP Monitoring and Accountability Framework. Although plausible explanations might exist, we struggle to understand how countries reporting disparate levels of district level DTP3 coverage, eg, <50% alongside coverage >100%, are ascribed achievement of a certain level of reasonable data quality (ie, “valid”) used in the GVAP Monitoring Framework to assess global progress. The results described here, albeit based on a fraction of countries who were willing to share subnational coverage data, suggest that the classification of countries’ district data as “valid” on the basis of national coverage estimates – either WUENIC≥90% or WUENIC identical to reported administrative coverage – may be insufficient and that the current approach might be reconsidered.

We also believe that further consideration may be warranted to collect information on the proportion of districts in each country that have reported district coverage >100%. At present, countries report the total number of districts with coverage ≥80% using discrete categories 80-89%, 90-94% and ≥95% and these counts are used towards the coverage indicator goal assessment if the district data are “valid”. There is an inherent assumption being made in the assessment framework: that the district coverage values ≥80% fall between 80% and 100% (inclusive). Our results show that this is not the case, with many districts reporting nonsensical coverage levels >100% – which should raise questions about the data underlying the determination of coverage levels and might impact the number of countries classified as having achieved the GVAP coverage target of ≥90% national DTP3 coverage and ≥80% DTP3 coverage in all districts. Moving forward, and with full recognition that district coverage data may be compromised at all coverage levels, not only at coverage levels >100%, we recommend differentiating between coverage values that are plausible (95-100%) vs those that are not (>100%) on Sheet 6 of the JRF.

## CONCLUSIONS

Progress against achievement of national target coverage can be monitored using annually available WUENIC estimates. Assessment of progress towards the district level GVAP coverage indicators, however, is limited by the availability of district level coverage data, “valid” or not. In 2017, 86 countries, or 44% of the 194 countries included in the GVAP assessment, did not report district level coverage data. Continued efforts to encourage countries to report coverage at the district or second subnational level are required.

Administrative district level coverage data are of unknown and varying quality in many countries – impacting the trustworthiness of data. Greater attention and political will are needed to improve frontline, point-of-service recording practices [12] in both facility-based and home-based records and in reporting processes at all levels, from the frontline to district level and up to national level. In some instances, the problems in subnational administrative data quality can be ascribed to poor subnational denominator data stemming from dated census information, poorly conducted censuses or other related, yet unidentified reasons. While problems in denominator data of administrative vaccination coverage cannot be ruled out, these data are not under the control of national immunization programmes. It is important for immunization programmes to ensure their own data are tidy and in good form. For both numerator and denominator data, data review tools and technical expertise exist to support the conduct of periodic comprehensive data reviews and data quality audits alongside targeted district level coverage surveys to assess how well coverage derived from administrative recording and reporting systems reflect programme performance on the frontlines. We believe our progress towards better understanding, documenting and ultimately improving district coverage estimates is not constrained by tools and technology; but hindered by a lack of commitment to improve practices and address processes that have been neglected for far too long.

In the interim, we must identify better strategies for accurately monitoring progress against GVAP vaccination coverage indicators. At present, it is not clear that the GVAP coverage indicator related to district level coverage achievement and the definition of “valid” district level coverage data are performing as intended and thus should be reconsidered. We look forward to an open dialogue with national immunization programmes and partners to search for alternative approaches.
