# Supplementary material for: Definition and use of “valid” district level vaccination coverage to monitor Global Vaccine Action Plan (GVAP) achievement: evidence for revisiting the district indicator
Source: J Glob Health. 2018 Jul 3;8(2):020404. doi: 10.7189/jogh.08.020404 (PMC6030671; doi:10.7189/jogh.08.020404)
Supplement: Online Supplementary Document [file jogh-08-020404-s001.pdf]

Online Supplementary Document

Brown. Definition and use of "valid" district level vaccination coverage to monitor Global Vaccine Action Plan (GVAP) achievement: evidence for revisiting the district indicator

J Glob Health 2018;8:020404

Appendix S1.

Below is a listing of countries to accompany the graphical display of the number of countries with agreement in the district coverage comparison in Figure 1. The country ISO-3 code is noted in parentheses next to each country name.

No district line list coverage data

Afghanistan (AFG), Algeria (DZA), Andorra (AND), Armenia (ARM), Antigua (ATG), Australia (AUS), Austria (AUT), Belgium (BEL), Bulgaria (BGR), Bahrain (BHR), Bahamas (BHS), Belarus (BLR), Barbados (BRB), Canada (CAN), China (CHN), Cook Islands (COK), Croatia (HRV), Cyprus (CYP), Czech Republic (CZE), Djibouti (DJI), Dominica (DMA), Denmark (DNK), Egypt (EGY), Estonia (EST), Finland (FIN), Fiji (FJI), France (FRA), Federated States of Micronesia (FSM), Germany (DEU), Georgia (GEO), Greece (GRC), Ireland (IRL), Iran (IRN), Iraq (IRQ), Iceland (ISL), Israel (ISR), Italy (ITA), Jordan (JOR), Japan (JPN), Kazakhstan (KAZ), Kyrgyzstan (KGZ), Kiribati (KIR), St Kitts and Nevis (KNA), Republic of Korea (KOR), Kuwait (KWT), Lebanon (LBN), Libya (LBY), Lithuania (LTU), Luxembourg (LUX), Latvia (LVA), Morocco (MAR), Monaco (MCO), Marshal Islands (MHL), Malta (MLT), Montenegro (MNE), Mongolia (MNG), Malaysia (MYS), Nigeria (NGA), Niue (NIU), Netherlands (NLD), Nauru (NRU), New Zealand (NZL), Philippines (PHL), Palau (PLW), Papua New Guinea (PNG), Poland (POL), Portugal (PRT), Qatar (QAT), Russia (RUS), Saudi Arabia (SAU), Sudan (SDN), Singapore (SGP), Samoa (WSM), San Marino (SMR), Serbia (SRB), Slovakia (SVK), Slovenia (SVN), Solomon Islands (SLB), Somalia (SOM), Spain (ESP), Sweden (SWE), Switzerland (CHE), Syria (SYR), Thailand (THA), Tajikistan (TJK), Turkmenistan (TKM), Tonga (TON), Tunisia (TUN), Turkey (TUR), Tuvalu (TUV), Ukraine (UKR), United Arab Emirates (ARE), United Kingdom of Great Brittan and Northern Ireland (GBR), United States of America (USA), Uzbekistan (UZB), Viet Nam (VNM), Vanuatu (VUT), Yemen (YEM)

District line list coverage data available

Zero (0) categories of agreement

Brazil (BRA), Chile (CHL), Ethiopia (ETH), Guatemala (GTM), Haiti (HTI), India (IND), Peru (PER), Suriname (SUR), Trinidad and Tobago (TTO)

One (1) category of agreement

Argentina (ARG), Bosnia and Herzegovina (BIH), Cambodia (KHM), Democratic Republic of Congo (COD), Dominican Republic (DOM), Equatorial Guinea (GNQ), Mozambique (MOZ), Namibia (NAM), Venezuela (VEN)

Two (2) categories of agreement

Cameroon (CMR), Cuba (CUB), Ecuador (ECU), Guinea (GIN), Honduras (HND), Moldova (MDA), Nepal (NPL), Senegal (SEN)

Three (3) categories of agreement

Burkina Faso (BFA), Belize (BLZ), Congo (COG), Laos PDR (LAO), Madagascar (MDG), Mali (MLI), Mauritania (MRT), Mauritius (MUS), Mexico (MEX), Norway (NOR), People's Republic of Korea (PRK), Paraguay (PRY), Romania (ROU), South Africa (ZAF), Sri Lanka (LKA), Tanzania (TZA), Uruguay (URY)

Four (4) categories of agreement

Azerbaijan (AZE), Brunei (BRN), Guyana (GUY), Hungary (HUN), Indonesia (IDN), Maldives (MDV), Myanmar (MMR), St Lucia (LCA), South Sudan (SSD), Uganda (UGA)

Five (5) categories of agreement

Angola (AGO), Albania (ALB), Burundi (BDI), Benin (BEN), Bangladesh (BGD), Bolivia (BOL), Bhutan (BTN), Botswana (BWA), Central African Republic (CAF), Chad (TCD), Cote d'Ivoire (CIV), Colombia (COL), Comoros (COM), Cabo Verde (CPV), Costa Rica (CRI), El Salvador (SLV), Eritrea (ERI), Gabon (GAB), Gambia (GMB), Ghana (GHA), Guinea Bissau (GNB), Grenada (GRD), Jamaica (JAM), Kenya (KEN), Liberia (LBR), Lesotho (LSO), Macedonia (MKD), Malawi (MWI), Niger (NER), Nicaragua (NIC), Oman (OMN), Pakistan (PAK), Panama (PAN), Rwanda (RWA), Sao Tome and Principe (STP), Seychelles (SYC), Sierra Leone (SLE), Swaziland / eSwatini (SWZ), Togo (TGO), Timor-Leste (TLS), St Vincent and the Grenadines (VCT), Zambia (ZMB), Zimbabwe (ZWE)
